# Crosstalk of Astrocytes and Other Cells during Ischemic Stroke

**DOI:** 10.3390/life12060910

**Published:** 2022-06-17

**Authors:** Tingting He, Guo-Yuan Yang, Zhijun Zhang

**Affiliations:** 1 Department of Neurology, Shanghai Tenth People’s Hospital, Tongji University, Shanghai 200072, China; htt199121@163.com; 2 Neuroscience and Neuroengineering Center, Med-X Research Institute and School of Biomedical Engineering, Shanghai Jiao Tong University, Shanghai 200030, China

**Keywords:** astrocyte, crosstalk, gliotransmitter, regeneration, stroke

## Abstract

Stroke is a leading cause of death and long-term disability worldwide. Astrocytes structurally compose tripartite synapses, blood–brain barrier, and the neurovascular unit and perform multiple functions through cell-to-cell signaling of neurons, glial cells, and vasculature. The crosstalk of astrocytes and other cells is complicated and incompletely understood. Here we review the role of astrocytes in response to ischemic stroke, both beneficial and detrimental, from a cell–cell interaction perspective. Reactive astrocytes provide neuroprotection through antioxidation and antiexcitatory effects and metabolic support; they also contribute to neurorestoration involving neurogenesis, synaptogenesis, angiogenesis, and oligodendrogenesis by crosstalk with stem cells and cell lineage. In the meantime, reactive astrocytes also play a vital role in neuroinflammation and brain edema. Glial scar formation in the chronic phase hinders functional recovery. We further discuss astrocyte enriched microRNAs and exosomes in the regulation of ischemic stroke. In addition, the latest notion of reactive astrocyte subsets and astrocytic activity revealed by optogenetics is mentioned. This review discusses the current understanding of the intimate molecular conversation between astrocytes and other cells and outlines its potential implications after ischemic stroke. “Neurocentric” strategies may not be sufficient for neurological protection and recovery; future therapeutic strategies could target reactive astrocytes.

## 1. The Way of Astrocyte Crosstalk with Other Cells

Astrocytes are the most abundant glial cell type; they are traditionally considered a kind of “supporting cell” and play a major role in maintaining homeostasis. Astrocytes have long been thought of as “passive” cells, implying that they just “listen” but never “talk”. However, emerging evidence demonstrates that astrocytes are also active participants in brain activity and are essential for cell–cell communication in the neural tissue. During ischemic stroke, the brain undergoes drastic changes and homeostasis breakdown, leading to severe injury. The role of astrocytes during this process is highly complex. Astrocytes show both beneficial and deleterious roles depending on different timepoints, different regions, and different aspects of stroke pathology. Exploring the communication of astrocytes and other cells during different stages of ischemic stroke and its influence on stroke outcomes is of great significance. This review paper discusses the recent advances in the study of astrocytes’ crosstalk with other cells in spatial and temporal dynamics under ischemic insults based on results from experimental animal studies.

Astrocytes have the structural basis for modulating homeostasis in larger brain regions and crosstalk with various cells as follows.

First, astrocytes are morphologically complex and branched with numerous fine processes, which envelop and directly contact with almost all parenchymal cells, including neurons, microglia, oligodendrocytes, endothelial cells, and immune cells, in the central nervous system [1]. Astrocytes dynamically change in response to alterations in their environment. Astrocytes can rapidly extend and retract fine processes to engage and disengage from motile postsynaptic dendritic spines with higher average motility than their dendritic spine counterparts [2].

Second, astrocytes are extensively coupled into homocellular or heterocellular networks via gap junction channels. Intracellular calcium changes in astrocytes can propagate to fine processes and other glial cells through gap junctions [3,4]. The calcium dynamics convey powerful signals due to their influence on protein kinases, ion channels, and vesicular release. Therefore, astrocytes can function as a syncytium of interconnected cells [5]. 

**Figure 1:**
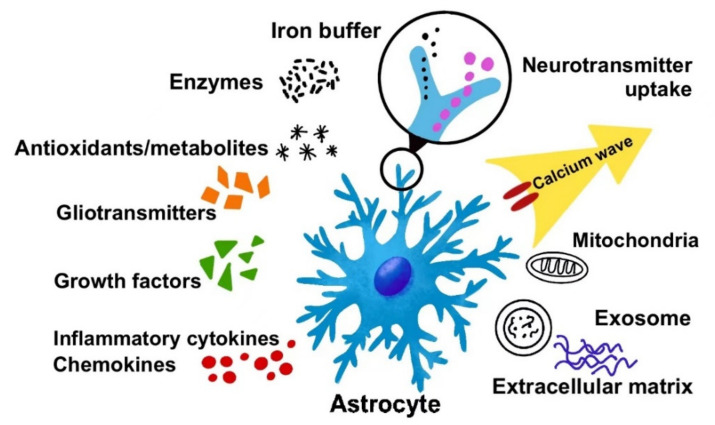
The major ways of astrocytic cell–cell communication and microenvironment regulation. Reactive astrocytes secrete a wide range of factors modulating the microenvironment and communicating with other cells, including gliotransmitters (glutamate, ATP, and D-serine), growth factors (e.g., BDNF, GDNF), inflammatory cytokines (e.g., interleukins, TNF-α, TGF-β), chemokines (e.g., CXCL12), metabolites (e.g., lactate), and enzymes (e.g., MMPs). Apart from small molecules, they can even send mitochondria and exosomes to other cells to convey messages. Astrocytes secrete extracellular matrix, which is a major component of the microenvironment. The processes of astrocytes can uptake neurotransmitters (e.g., glutamate, GABA) and buffer irons (e.g., Ca^2+^, K^+^) to maintain homeostasis of the microenvironment and influence synaptic plasticity. Despite secreting factors, there are calcium signals quickly propagating through gap junctions formed by connexins between astrocytes and other cells in order to coordinate cell functions.

Third, astrocytes express a large repertoire of receptors, responding to all neurotransmitters, neuromodulators, hormones, growth factors, chemokines, and steroids by changing cytosolic Ca^2+^ or cAMP [6], which gives astrocytes the ability to detect microenvironment changes. They also release glutamate, D-serine, ATP [7], GABA [8], prostaglandins, and neuropeptides, generally called “gliotransmitters”. These gliotransmitters have been shown to modulate other glial, neuronal, or vascular cells [9]. In addition, a wide range of factors is secreted by astrocytes to modulate microenvironments. Astrocyte-derived exosomes are also one of the most significant ways of communication between astrocytes and surrounding cells [10]. The major ways of cell communication and microenvironment regulation by astrocytes are shown in Figure 1.

The understanding of astrocytes has increased considerably over the past two decades owing to new technological advances in transcriptomics, in vivo imaging, optogenetics, and chemogenetics. The diversity and complexity of astrocytic contribution to health and disease are being unveiled, challenging the “neurocentric” dogma. Optogenetics is a useful technique; it allows noninvasive manipulation with high specificity and temporal precision on a millisecond scale [11]. In general, channelrhodopsin-2 (ChR2), calcium-translocating channelrhodopsin (CatCh), ChETA, and LiGluR are used for the depolarization of the membrane. Light-driven outward proton pumps such as archaerhodopsin (Arch) and chloride pumps such as halorhodopsin (NpHR) can induce hyperpolarization of the membrane after photostimulation [12]. Optogenetics has been mainly used to manipulate neuronal activity to investigate neural circuits [13]. Optogenetic approaches can also selectively manipulate astrocytic activity with specific promoters such as GFAP or Mlc1. Astrocyte-specific opsin expression in vivo is achieved by injecting an adeno-associated virus or lentivirus encoding an astrocyte-specific opsin into a target region. Alternatively, the opsin can be expressed using Cre/loxP and tetO-tTA systems in a genetically engineered mouse line [14]. Thus, optogenetic targeting of astrocytes provides a robust experimental model to elucidate the role of astrocytes in brain functions. 

Signals from optogenetically modulated astrocytes can drive neuronal activity and animal behavior. Glial photostimulation can lead to perturbation of motor behavior in the cerebellum. The underlying mechanism is that cerebellar astrocyte stimulation leads to glutamate release which then activates AMPA receptors on Purkinje cells and mGluR1 on synapses of parallel fibers to Purkinje cells. Then LTD is induced and motor behavior is changed. This finding indicates that astrocytic activity can modulate neuronal activity, synaptic plasticity, and behavioral response [15]. Optogenetic stimulation of ChR2-expressing astrocytes in the brain stem chemoreceptor areas can trigger robust respiratory responses via ATP-dependent mechanism in vivo [16]. Optogenetically activated astrocytes affect retrotrapezoid nucleus neurons via an ATP-dependent manner, while in the locus coeruleus, astrocytes activate NAergic neurons by releasing glutamate. So, there exists an area-specific and transmitter-dependent manner of astrocytic modulation of neuronal activity.

Optogenetic activation of astrocytes in the mouse posterior hypothalamus increases both rapid eye movement sleep (REM) and non–rapid eye movement sleep (NREM) during the active phase of sleep–wake regulation [17]. Interestingly, selective photostimulation of astrocytes in the anterior cingulate cortex increased the wakefulness and disturbance of NREM under neuropathic pain condition [18]. Thus, optogenetic manipulation of astrocytes in specific brain regions has different effects on sleep. This phenomenon may be due to astrocytic adenosine release and the different distribution of wake- and sleep-active neurons [19]. Using electrophysiological recording and two-photon imaging, a study showed that astrocytes could trigger a switch of the cortical circuit to the slow-oscillation-dominated state in the neocortex, and this was due to transient glutamate release from activated astrocytes [20]. This work not only directly demonstrated glutamate release by astrocytes after stimulation but also indicated that astrocytes could control the cortical synchronizations which were important for sleep and memory. Optogenetic stimulation of astrocytes localized in the medial basal hypothalamus could suppress food intake through increased extracellular levels of adenosine in a frequency-dependent manner, providing new insight into astrocytes in the control of energy states [21]. 

Optogenetic manipulation of astrocytes provides direct evidence for the active role of astrocytes at the circuit level; the communication between astrocytes and neurons not only plays a role in regulating synaptic function but also plays a role in dominating the activity of the neural network [22]. These studies can open up avenues for studying the role of astrocytes in higher-order brain functions and show that optogenetics is a good way of exploring astrocytic communication with other cell types. 

## 2. Functions of Astrocytes in Ischemic Stroke 

Stroke, of which approximately 87% is ischemic, is a leading cause of death and disability globally. Due to a lack of glucose and oxygen because of the loss of blood flow, neural tissue is biochemically and metabolically compromised, resulting in cell death after ischemic stroke. The role of astrocytes in ischemic stroke is complex; they work as versatile players in regulatory processes depending on context, region, and time.

### 2.1. Reactive Astrogliosis

Astrocytes undergo a dramatic morphology change which is commonly referred to as reactive astrogliosis after ischemic insult. Sofroniew gave a comprehensive and accurate definition of astrogliosis as “a finely gradated continuum of progressive changes in molecular expression, cellular hypertrophy, proliferation, and scar formation, which are subtly regulated by complex intercellular and intracellular signaling” [23]. A consensus statement by various researchers recently defined reactive astrocytes as “astrocytes engage in molecularly defined programs involving changes in transcriptional regulation, as well as biochemical, morphological, metabolic, and physiological remodeling, which ultimately result in the gain of new function(s) or loss or upregulation of homeostatic ones in response to pathology” [24].

After brain ischemia, astrocytes changed from the normally bushy form to a hypertrophic stellate shape and then to a highly polarized shape with long processes pointing to the ischemic core [25]. Reactive astrocyte proliferation was increased, marked by upregulated GFAP in the acute phase (days 1–4 post-ischemia). Polarized astrocytes with elongated processes were steadily increased from the subacute phase (days 4–8 post-ischemia) and gradually formed a mature glial scar until the chronic phase (days 8–14 post-ischemia), also shown by high-resolution imaging in the ischemic cortex in vivo [26]. Reactive astrocytes have heterogeneity in their sensitivity to ischemia, distance to the ischemic core, and subtypes [27]. 

An acute increase in astrocytic Ca^2+^ signaling and subsequent glutamate and GABA release may represent the initial step after ischemia; Ca^2+^ can regulate many downstream signaling intermediates such as the phosphatase calcineurin, which will then activate NFAT or N-cadherin pathways [28]. The STAT3, p38 MAPK, nuclear factor κB (NF-κB), and transforming growth factor-beta (TGF-β) signaling pathways are involved in inducing the production of GFAP and transcription factors or retro-inhibitors of other pathways (e.g., SOCS3 for the JAK-STAT3 pathway or IκB for the NF-κB pathway) [29]. The STAT3 pathway seems to play a prominent role in shaping the transcriptome of reactive astrocytes. Epidermal growth factor (EGF), fibroblast growth factor (FGF), endothelin-1, and ATP are reported to contribute to the proliferation of reactive astrocytes [30,31,32]. The proliferation of reactive astrocytes is also regulated by Notch-1 in the peri-infarct region [33]. In addition, class B scavenger receptor CD36 has recently been reported to be involved in astrocyte activation and glial scar formation in ischemic stroke patients [34]. 

Reactive astrogliosis was traditionally considered to form glial scars that hamper neuronal repair. Nevertheless, increasing evidence indicated that reactive astrocytes could also exert beneficial functions. Transgenic ablation of reactive astrocytes after CNS injury markedly increased neuronal death and exacerbated tissue degeneration [35]. There is a strong interest in better understanding this specific transformation of astrocytes in response to stroke. 

### 2.2. Functions of Reactive Astrocytes in the Pathogenesis of Stroke 

#### 2.2.1. Astrocyte-Neuron Interaction: Neuroprotection after Stroke

*Metabolic support:* Glycogen is predominantly stored in astrocytes and serves as the main energy reserve in the brain. During an energy crisis such as ischemia insult, glycogen can be broken down to glucose, metabolized to pyruvate via glycolysis, and eventually converted to lactate. Then, lactate is transported from astrocytes to neurons as an energy substrate through monocarboxylic acid transporters (MCTs) [36]. Thus, reactive astrocytes can function as an energy source to supply neurons with substrates. Stroke stimulation induces astrocyte-specific MCT4 expression and enhances lactate production. Astrocyte-derived lactate may be critical for the survival of vulnerable neurons and contribute to stroke prevention. However, diminished lactate supply from astrocytes could facilitate stroke-induced neurodegeneration [37]; further study will be needed to address this question.

*Antiexcitatory effect:* Ischemic injury caused by excess glutamate is a decisive factor in stroke outcome. Failure of astrocytes to remove this excess glutamate leads to excitotoxic damage. Excess glutamate in the ischemic brain causes hyperactivation of ionotropic glutamate receptors, which induces Ca^2+^ influx into the neurons and then triggers neuronal cell death [38]. Astrocytes play an important role in glutamate regulation through involvement in the glutamate–glutamine cycle, namely by clearing synaptic glutamate and converting it into glutamine which will finally be transferred back into neurons. In this way, astrocytes have an antiexcitatory effect by uptaking glutamate with glutamate transporters EAAT1 (GLAST) and EAAT2 (GLT-1) [39]. Upregulation of GLT-1 decreases infarct size and promotes behavioral recovery [40]. Impairment of astrocyte activation decreased glutamate uptake and increased infarct volume in the GFAP^−/−^Vim^−/−^ mouse model of stroke [41]. The glutamate–glutamine cycle interfaces with several metabolic pathways such as the tricarboxylic acid (TCA) cycle and even lipid signaling [42]. A recent study showed that synaptic lipid signaling can modulate glutamatergic transmission. The enzyme autotaxin in astrocytic presynaptic processes synthesizes lysophosphatidic acid 2 (LPA2) and influences glutamatergic transmission via presynaptic LPA2 receptors. Astrocyte-specific deletion of autotaxin reduces cortical excitability and subsequent negative stroke outcome [43].

*Antioxidant function:* Astrocytes play a crucial role in antioxidant defense. Astrocytes contain the highest concentration of antioxidants and provide neighboring neurons with substrates for antioxidants such as glutathione [44]. Glutathione is also involved in the glutamate–glutamine cycle and is increased in neurons after being co-cultured with astrocytes [45]. Glutathione depletion exacerbated cortical infarction and edema after ischemia. Astrocytes from transgenic mice with low glutathione levels were ineffective at protecting neurons from apoptotic cell death [46]. Thus, glutathione loss is involved in the development of infarction. Reactive astrocytes may also produce various free radicals in response to ischemia. The increased expression of S-100β and inducible nitric oxide synthase (iNOS) led to oxidative stress [47,48], which mediated neuronal death after stroke.

*Mitochondria transfer:* Mitochondria represents intracellular cores for energetics and cell viability; they can be released and transferred to other cells under certain conditions [49]. For example, retinal neurons may transfer damaged mitochondria to astrocytes for disposal or recycling in wild-type mice [50]. This prominent finding suggested that astrocytes may release mitochondrial particles that enter adjacent neurons after transient focal cerebral ischemia in mice. This transfer amplified cell survival signals mediated by CD38 and cyclic ADP ribose signaling in a calcium-dependent manner, and it eventually supported cell viability and functional recovery after stroke [51]. Astrocytic “donation” of functional mitochondria to neurons is a novel endogenous neuroprotective and neuronal recovery mechanism after stroke and a potential mode of astrocyte–neuron crosstalk.

*Exosomes:* Recent studies also showed that astrocytes sent out exosomes, also called extracellular vesicles, which could transfer a large diversity of molecules such as lipids, nucleic acids, and proteins, serving as a new platform for complex intercellular communication [52,53]. A previous study showed that astrocyte-derived exosomes could increase neuronal cell survival under ischemic conditions [54]. The mechanisms have been revealed recently. Astrocytes also shuttle miR-190b through exosomes to inhibit neuronal apoptosis through modulating autophagy [55]. Exosomes from ischemic preconditioned astrocytes shuttled miR-92b-3p to protect neurons against oxygen and glucose deprivation in vitro [56]. Astrocytic exosome-conveyed microRNA-34c is neuroprotective via TLR7 and NF-κB/MAPK pathways against cerebral ischemia/reperfusion injury in vivo [57]. Astrocyte-derived exosomal *miR-361*, which downregulates the AMPK/mTOR signaling pathway by targeting CTSB, is neuroprotective both in vitro and in vivo [58]. Besides conveying miRNAs, exosomes also transfer proteins. Extracellular vesicles secreted by astrocytes transport apolipoprotein to neurons and mediate neuronal survival upon oxidative stress [59]. Astrocyte-derived exosomes treated with a semaphorin 3A inhibitor enhance stroke recovery via prostaglandin D2 synthase [60]. However, this protective role of astrocytic exosomes exists under certain conditions; another study revealed that astrocytic exosomes in response to inflammatory stimulus interleukin-1β (IL-1β) contained cargo microRNAs and proteins that reduced neurite outgrowth and neuronal firing and promoted neuronal apoptosis [61]. Activated human primary astrocyte-derived extracellular vesicles tested by label-free quantitative proteomic profiling revealed a notable upregulation of proteins including actin-associated molecules, integrins, and major histocompatibility complex in IL-1β-treated groups, they could be uptaken by neurons and thus negatively modulate neuronal uptake, differentiation, and firing [62]. Identification of astrocyte-derived exosomes’ effects on both short- and long-distance targets and strategies may lead to the finding and development of new diagnostic and therapeutic methods.

*Other mechanisms:* Astrocytes can also release several neuroprotectants, including erythropoietin (EPO), VEGF, and glial-derived neurotrophic factor (GDNF), all of which can reduce ischemic neuronal damage after stroke [63,64,65]. Reactive astrocytes are known as an important source of steroids, especially estrogen. The expression of the enzyme aromatase and its production of 17β-estradiol in astrocytes is upregulated following brain ischemia. Knockout of aromatase in astrocytes in mice, leading to downregulation of estrogen and attenuated astrogliosis, displayed enhanced neuronal damage under ischemic conditions [66]. Interestingly, neurons also secrete estrogen 17β-estradiol, which is also critical for astrocyte activation and release of neurotrophic factors in the ischemic brain [67]. Despite releasing factors, a subset of reactive astrocytes within the ischemic penumbra region is found to be transformed into phagocytic cells. Phagocytic astrocytes upregulate ABCA1 and its downstream pathway molecules including MEGF10 and GULP1 to engulf large neuronal debris contributing to the remodeling of damaged tissues [68].

#### 2.2.2. Astrocyte-Astrocyte Interaction: Functions of Gap Junctions after Stroke

Connexins (CXs), a kind of gap junction channel, allow for direct electrical and biochemical communication and support astrocytic key functions such as the spreading of metabolic substrates, spatial K^+^ buffering, and calcium waves [69]. Hemichannels are precursors of gap junctions existing in the plasma and may open in response to triggers such as cell depolarization and calcium change; they may also function as gliotransmitter release channels [70,71]. Hypoxia/reoxygenation and metabolic starvation lead to the opening of hemichannels in cultured astrocytes [72]. Seven different CXs exist in different cell types: Cx43 is mainly expressed in astrocytes; Cx36 is the main neuronal connexin; and oligodendrocytes express Cx32, Cx47, and Cx29 [73,74]. These channels have distinct functions in different cell types, and their expression can change dramatically under different conditions [74]. 

The role of astrocytic gap junctions in stroke remains controversial. Astrocytic gap junctions remain open following ischemia in vivo and hypoxia in vitro [75,76]; they could increase infarct volume by spreading proapoptotic factors, which caused delayed cell death in the penumbral region [77]. ATP channeling, calcium wave spreading, and propagation of neuronal activity depression may also be implicated in the expansion of infarction [78]. Studies showed that gap junction blockers reduced neuronal death and infarct volume in different experimental stroke models [79]. Astrocytic Cx43 activation played a critical role in neuronal death by NMDA neurotoxicity during inflammation in neuron/astrocyte co-cultures. This excitotoxicity could be inhibited by a Cx43 blocker or Cx43 knockout in astrocytes [80]. Inactivation of mGluR2 reduced the increased expression of Cx36 and neuronal death after ischemia. Genetic elimination of Cx36 also reduced neuronal death after ischemia [81]. Thus, Cx36 is a critical component of glutamate-dependent neuronal death. Excessive opening of Cx43 hemichannels pathologically contributed to cell swelling and death by osmotic shifts as well as calcium overload [82]. However, global heterozygous genetic ablation of Cx43 increased the infarct size and number of apoptotic cells in the penumbra area of ischemic mice with reduced astrogliosis [83]. A similar outcome was obtained in a follow-up investigation using specific astrocyte Cx43 knockout mice [84]. 

The reason for the controversy remains elusive. However, the different functions of gap junctions and their hemichannels should be taken into consideration; the blocking reagents and genetic modification have differences in targets, selectivity, mechanisms, and efficacy. Connexin mimetic peptides can block hemichannels without blocking gap junctions. Gap26/27, which mimics Cx43, was proved to be cardioprotective against infarction [85]. The role of these mimics in ischemic brain injury needs to be investigated in the future. The phosphorylation of Cx43, which influences its internalization, degradation, and hemichannel activity, should not be overlooked [86]. Moreover, CXs have both channel functions and nonchannel functions; many CXs can be anchored to scaffolding proteins via C-terminal (CT) interaction and influence gene expression [87]. The impact of CT truncation of Cx43 includes increased infarct volume, reduced astrogliosis, and more microglial infiltration in the MCAO model [88]. The nonchannel functions complicate its role after ischemic injury.

#### 2.2.3. Astrocyte and Microglia Crosstalk after Stroke: Inflammation after Stroke

Inflammation has long been regarded as a critical contributor to the pathophysiology of ischemic stroke [89]. Both microglia and astrocytes are major components of the innate immune system in the brain and respond to damage-associated molecule patterns (DAMPs) after ischemic stroke; their bidirectional communication has recently been at the forefront of glial research. Microglia activation is the beginning of the inflammatory response, followed by infiltration of peripheral immune cells and astrocyte reactivity [90]. Early transcriptome studies revealed two gene expression patterns for two subtypes of astrocytes: an A1 neurotoxic phenotype after exposure to specific cytokines including IL-1α, TNF-α, and the complement component subunit 1q (C1q) secreted by microglia that were exposed to lipopolysaccharide, and an A2 neuroprotective phenotype predominant at 72 h after ischemic stroke [91,92]. These terminologies parallel the M1 and M2 types of activation in macrophages/microglia. A1 astrocytes show a compromised ability to induce synapse formation and phagocytose synapses which can induce neuronal apoptosis, and A2 astrocytes show upregulation of many neurotrophic factors and secrete proteins that promote CNS synaptogenesis, indicating neuroprotective and reparative functions [91]. Activated microglia can release a series of proinflammatory cytokines and chemokines. Microglia-derived cytokines can work as triggers and modulators of astrogliosis, because astrocytes express innate immune pattern recognition receptors (PRRs), such as toll-like receptors (TLRs), NOD-like receptors (NLRs), mannose receptors, scavenger receptors, and complement receptors [93]. The release of IL-1α, TNF-α, and also fragmented and dysfunctional mitochondria from microglia trigger the A1 astrocytic response [94]. C1q secreted by microglia also promotes A1 phenotype transformation, which is potentially mediated by scavenger receptor Megf10 expressed by astrocytes [95]. Microinjection of the recombinant IL-1 into the neonatal brain could induce astrogliosis. The IL-6 or IL-1β knockout mice showed less astrogliosis after injury compared with the WT mice [96,97]. Suppressing microglial proliferation with olomoucine could attenuate glial scar formation after injury in rats [98]. Microglial TNF-a production promotes astrocyte glutamate release, which boosts neuron excitotoxicity, so microglia also modulate excitatory neurotransmission mediated by astrocytes [99]. ATP derived from microglia could bind to P2Y1R located on the astrocyte membrane to amplify ATP release and increase excitatory postsynaptic currency frequency [100]. 

The role of astrocytes in local immune reactivity and inflammation has long been overlooked. Reactive astrocytes can also release gliotransmitters; proinflammatory mediators such as IL-6, TNF-α, IL-1α, IL-1β, and IFN-γ; and free radicals, which act on the receptor expressed microglia to create a paracrine/autocrine feedback loop [101]. A recent transcriptome analysis after stroke shows that markers of reactive astrocytes, *Lcn2*, *GFAP*, *vimentin*, and *Timp1*, were highly expressed and contributed to inflammation (e.g., *Spp1*, *Cd52*, *Lcn2*, and *Ifi202b*) [92]. Astrocytes can induce the increased expression of MCP-1/CCR2 in microglia after ischemic stroke [102]. TGF-β signaling is increased in reactive astrocytes and activates microglia after ischemic stroke [103]. Galectin-9 serves as a communication signal of astrocyte–microglia crosstalk and promotes microglial TNF-α secretion in the co-culture system of astrocytes and microglia. Recombinant galectin-9 increased TNF-α and IL-6 secretion from microglia [104]. Moreover, IL-10 released by microglia stimulates astrocytic TGF-β release, which in turn attenuates microglial activation as a feedback loop [105]. ATP released from astrocytes after traumatic brain injury activates microglial cells, which could be inhibited by blockers of G protein-coupled purinergic receptors and connexin channels. Astrocytes secrete lipocalin protein orosomucoid-2 (ORM2) upon inflammatory stimulation, which modulates microglial activation. ORM2 can bind with microglial C-C chemokine receptor type 5 (CCR5) and block the chemokine C-X-C motif ligand (CXCL)-4–CCR5 interaction that is critical for microglial activation to exert anti-inflammatory effects during brain inflammation [106]. A recent study revealed that specifically depleting astrocyte-derived estrogen after global cerebral ischemia led to upregulation of A2 astrocytes and less microglial activation, which can be rescued by exogenous 17β-estradiol administration [66]. This implies that astrocytic steroids can modulate microglial function. Astrocytes also secrete high levels of another lipocalin protein, LCN2, revealed by recent transcriptome analyses one day after experimental ischemic stroke, whose receptor LCN2R, mainly expressed in microglia and neurons, opposes ORM2 functions and enhances microglial activity in vascular dementia animals [107]. Astrocyte-derived exosomes conveying Cox2 small interfering RNA could restore microglial phagocytic activity after being uptaken by microglia in a neurodegenerative model [108]. These results suggested that astrocytic molecule release and purinergic signaling are important modulators of inflammatory responses. Briefly, microglia- and astrocyte-derived factors can regulate each other. However, current studies on the microglia-astrocyte crosstalk are still mainly focused on CNS inflammatory diseases, and future research is still needed.

Recent findings suggested that astrocytes also interact with other infiltrating peripheral immune cells after stroke to modulate post-stroke neuroinflammation [109]. The ablation of IκB in astrocytes reduced peripheral immune cell infiltration into the CNS in the experimental autoimmune encephalomyelitis (EAE) model [110]. These results indicated that reducing the astroglial NF-κB signaling pathway would attenuate proinflammatory cytokines produced by T cells during acute disease. Astrocytes enhanced lymphocyte toxicity after ischemic stroke by activating cytotoxic functions of natural killer cells (NKs) and CD8^+^ T lymphocytes mediated by IL-15/IL-15 receptor-α. IL-15 knockdown in astrocytes resulted in a decrease in tissue damage and better neurological outcomes after stroke [111]. Astrocytes also act as a partial source of IL-17, which interacts with TNF-α and thus leads to neutrophil invasion and the expression of various proinflammatory molecules in vivo [112]. Astrocytes increase IL-33 and CCL1 levels in response to stroke, and IL-33 is thought to promote the proliferation of Treg cells after stroke [113]. 

These studies suggest that the global outcome is the result of intensive crosstalk between astrocytes, microglia, and infiltrating immune cells in CNS injury. 

#### 2.2.4. Astrocyte and Endothelial Crosstalk: BBB Integrity and Edema after Stroke

The critical importance of astrocytes in the induction and maintenance of BBB structure and function has long been established. A recent study gives direct evidence by adopting a tamoxifen-inducible astrocyte ablation mouse model; leakage of fluorescently labeled cadaverine and blood plasma fibrinogen into the brain has been detected in adult mice [114]. Conditional knockout of astrocytic Wnt release led to brain edema and increased vascular tracer leakage [115]. Astrocytes could synthesize canonical tight junction proteins claudin 1, claudin 4, and junctional adhesion molecule-A [116]. Treatment with extracellular vesicles from healthy astrocytes increased transendothelial electrical resistance and upregulated expression of tight junction proteins in monolayers of human brain endothelial cells in vitro [117]. Astrocytes can also adjust the cerebrovascular tone to coordinate local blood supply with neuronal activity changes and local metabolic demands, forming a “neuroglia-vascular unit” [118]. Optogenetic stimulation of cortical astrocytes elicits a widespread increase in cerebral blood flow [119].

BBB breakdown leads to brain edema and hemorrhagic transformation, which are major complications during acute ischemic stroke. Astrocytes act as key regulators of brain edema, and their endfeet are estimated to ensheath more than 99% surface of blood vessels [120]. The earliest and prominent astrocytic response to ischemia is astrocyte swelling which occurs at endfeet around capillaries due to ionic and osmotic dysfunction [121]. Astrocytes play a major role in cytotoxic edema, particularly via water channel aquaporin 4 (AQP4), which is highly expressed on astrocyte endfeet. AQP4-depleted mice were observed to have enhanced neurologic outcomes and reduced brain edema after focal ischemia [122]. Furthermore, AQP4 is also involved in astrocyte migration, glial scar formation, neuroinflammation, and extracellular K^+^ uptake [123]. Loss of astrocyte–endothelial contacts resulted in the breakdown of BBB integrity leading to vasogenic edema during ischemia [124]. Astrocyte-secreted MMPs and VEGF also increase blood vessel permeability and vasogenic edema after stroke [125]. The increased expression of VEGF-A in reactive astrocytes led to disputed BBB integrity by downregulating claudin-5 and occludin in endothelial cells [126]. Neutralization of IL-9 could downregulate astrocyte-derived VEFG-A to protect BBB integrity [127]. However, reactive astrocyte-derived pentraxin-3 might support BBB integrity by regulating VEGF-related mechanisms in peri-infarct areas, which may comprise a compensatory mechanism [128]. FGF2 also plays a dual role in the regulation of endothelial barrier function. Autocrine secretion of FGF2 is necessary for the establishment of a tight endothelial barrier, whereas exogenous FGF2 in concentrations higher than 4 ng/mL does harm, implying it regulates endothelial barrier function in a concentration-dependent manner [129]. 

Pericytes are also important components of BBB and have receptors for a large number of vasoactive signaling molecules. Astrocytic laminin regulates pericyte differentiation and maintains BBB integrity [130]. The pericyte conditioned medium promotes astrocyte proliferation after PDGFB treatment. GFAP knockdown mice showed higher pericyte/endothelial cell ratios than those observed in wild-type mice [131]. However, direct evidence for astrocyte augmentation of pericyte coverage is lacking, and their communication under ischemic insult still needs further study.

#### 2.2.5. Astrocytic MicroRNAs in Stroke

MicroRNAs (miRNAs), 18–25 nucleotide-long noncoding RNAs, are potent post-transcriptional regulators of protein expression through interaction with specific mRNA targets [132]. This post-transcriptional control is very complex because miRNAs can bind multiple mRNAs. Meanwhile, mRNAs can be bound by multiple miRNAs. Numerous miRNAs have a preferential cellular expression pattern. According to a recent microarray analysis of miRNA expression in major cell types of the CNS, miR-181, and miR-29 appeared to be more highly expressed in astrocytes [133]. The miRNA profile changed after stroke, which suggested that miRNAs could contribute to ischemic injury by altering key signaling elements [134,135]. The fast post-transcriptional effect and multitarget characteristics provide miRNAs with greater therapeutic potential for stroke. Here we will discuss several miRNAs enriched in astrocytes. miR-210 is significantly upregulated in astrocytes and activated by hypoxia-inducible factor-alpha (HIF-1a) during hypoxic injury [136]. Our study found that blood miR-210 was significantly decreased in stroke patients for up to 14 days and correlated with clinical prognosis. The following animal study also confirmed the positive correlation between blood and brain after stroke. Thus, miR-210 acts as a novel sensitive biomarker in acute cerebral ischemia [137]. miR-181a and miR-29a are miRNAs that coordinate mitochondrial homeostasis. The antagomir of miR-181a could reduce infarct lesions and CA1 neuronal loss after ischemia in vivo and protect primary cultured astrocytes but not neurons after OGD in vitro [138].

miR-29a agomir upregulated PUMA, a member of the BCL2 family, to protect astrocyte and mitochondrial function [139]. Controversial findings have been detected in vivo; upregulation of miR-29a protected neurons from apoptosis during cerebral ischemia [140], while downregulation of miR-29 also rescued heart ischemia/reperfusion injury [141]. This controversy may be due to the different targets of miR-29 in different cells, because luciferase assays indicated that the miR-29 family targets BCL2 family members, both proapoptotic (BAK and PUMA) and antiapoptotic (BCL-w and MCL1) [142]. miR-29b is significantly downregulated and negatively associated with clinical severity in ischemic patients; a similar pattern is observed in mouse brains and blood. Dual-luciferase reporter system confirmed that AQP4 was the direct target of miR-29b. Overexpression of miR-29b decreased AQP4 expression, infarction volume and BBB disruption [143]. miR-146a is enriched in astrocytes and a potent regulator of the inflammatory response through interaction with TLR signaling [144]. miR-146a could suppress IRAK-1 and TRAF-6 to reduce the release of proinflammatory cytokines and subsequently protect liver ischemia/reperfusion injury [145]. Increased expression of miR-146a could decrease myocardial ischemia/reperfusion injury [146]. The miR-146aC > G polymorphism and miR-146aG/-149T/-196a2C/-499G allele combination were significantly associated with ischemic stroke prevalence in a clinical study [147]. Although astrocytic miRNAs could be potential therapeutic targets for the treatment of stroke through anti-inflammation or antioxidation, their safety and other limitations need further investigation. Astrocytic exosomes also convey miRNAs to regulate other cells, which has been discussed in another section.

## 3. Functions of Astrocytes in Post-Stroke Regeneration

### 3.1. Glial Scar Formation and MMP-9

A glial scar consists predominately of reactive astrocytes, microglia, and ECM. Highly proliferative “scar-forming” astrocytes located around lesions express specific transcripts such as chondroitin sulfate proteoglycans (CSPGs) and N-cadherin, while hypertrophic reactive astrocytes express several members of the β-catenin pathway such as Ctnnb [148]. A glial scar could isolate the ischemic lesion to protect surviving tissue from the harmful molecules; on the other hand, it has traditionally been viewed as a physical barrier for neurite outgrowth and axonal regeneration. The secreted inhibitory molecules, CSPGs, form an unfavorable environment for axonal outgrowth in the long term [149]. The decreased CSPG expression level could enhance axon growth in vitro [150]. Matrix metalloproteinase-9 (MMP-9) is detrimental in the acute phase but could be beneficial for recovery in the subacute phase of stroke by breaking down CSPGs [151]. Our group constructed a hypoxia response element-regulated MMP-9 vector to confine MMP-9 expression only in the hypoxic region; this vector promoted behavioral recovery after ischemia without aggravating BBB damage in the subacute phase of ischemia [152]. Immunosuppressive agent cyclosporine A significantly reduced astrogliosis and glial scar formation, implying glial scar formation could be modulated by inflammatory signaling. Microglia can also regulate glial scar formation; we found that M2 microglial extracellular vesicles conveying miR-124 could reduce glial scar formation via the STAT3 pathway after stroke [153]. Nevertheless, some researchers indicate that “not everything is scary about a glial scar” by the evidence that axons failed to regrow through regions depleted of reactive astrocytes in a spinal cord injury model [154]. 

### 3.2. Neurogenesis and Synaptogenesis: Astrocytes and Neuroblasts

Astrocytic processes enwrap synapses and form a physical barrier that limits the diffusion of the neurotransmitter. Thus, astrocytes form “tripartite” synapses together with presynaptic and postsynaptic terminals due to this intimate physical contact and sophisticated chemical regulation [155]. Reactive astrocytes can release several growth factors, such as NGF, BDNF, GDNF, VEGF, FGF2, and CNTF [24], which provide stem cells and other cells with appropriate factors for survival and neural repair. Ciliary neurotrophic factor (CNTF) is exclusively expressed in astrocytes; stroke induces upregulation of CNTF [156]. Ischemic stroke stimulates endogenous neurogenesis in the subventricular zone (SVZ) and dentate gyrus and subsequent migration of neuroblasts targeting ischemic lesions in the adult rodent brain [157]. Neurogenesis is abolished in CNTF knockout mice [158]. Astrocytic calcium waves in SVZ enhanced the self-renewal and migration capacity of neural stem cells (NSCs) and neural progenitor cells (NPCs) in a mouse stroke model and were mediated by the Notch signaling pathway [159]. Astrocytes in the ischemic striatum form a migratory scaffold of neuroblasts from SVZ to the ischemic region [160]. Reactive astrocytes around an ischemic lesion secreted chemokine CXCL12, which attracted neuroblast migration [161]. Our group found that AAV-mediated CXCL12 expression upregulated the proliferation of NSCs in SVZ and migration of neuroblasts to the peri-infarct region, thus promoting neurogenesis post-stroke [162]. Co-transplantation of astrocytes and NSCs into ischemic stroke rats resulted in the increased survival rate, proliferation, and neuronal differentiation of the transplanted NSCs compared with NSC transplantation alone [163]. 

Astrocytes are important players in the establishment of synaptic connectivity including control of synaptogenesis, synaptic plasticity (mentioned earlier), and synapse elimination [164]. Astrocytes are the major cellular source of IL-17A, which maintained and increased NPC survival and neuronal differentiation in SVZ and promoted subsequent synaptogenesis and functional recovery after stroke [165]. Thrombospondin (TSP) 1 and 2 secreted from astrocytes increased after brain ischemia and promoted synaptogenesis and axon sprouting in vivo [166]. Heterogeneity existed in the synaptogenic profile of astrocytes from different brain regions, which may be due to greatly varied expression of glypicans 4 and 6; hevin; and secreted protein, acidic and rich in cysteine (SPARC) [167]. 

Upregulation of the cholesterol-binding sigma-1 receptor in astrocytes is beneficial for axonal sprouting; a sigma-1 receptor agonist enhanced neurite outgrowth, promoting behavioral recovery after stroke [168]. A recent study showed that astrocytes can promote structural remodeling of striato-cortical circuits through the release of extracellular vesicles in the tMCAO mouse model [169]. A meta-analysis of astrocytic EV proteomes revealed that proteins which regulate axon outgrowth and guidance, including TUBB, ACTG1, RTN4, and Rab11A, are upregulated. However, upregulation of astrocytic ephrin-A5 blocked neuronal outgrowth and impaired behavioral recovery in the pMCAO mouse model, while inhibition of ephrin-A5 is beneficial [170]. L-lactate and L-2HG from astrocytes act on neuronal metabotropic GABAB receptors to boost cAMP signaling, thus promoting corticospinal tract axon regeneration in the adult mouse spinal cord [171]. Evidence of astrocytes mediating axon regeneration via metabolites in stroke is still awaiting further research.

### 3.3. The Stem Cell-Related Properties of Reactive Astrocytes

Glial fibrillary acidic protein (GFAP), an intermediate filament protein, is generally used as a marker to identify astrocytes. However, astrocyte-like NSCs in neurogenic niches also express GFAP. Subpopulations of reactive astrocytes proliferated and expressed stem cell-associated proteins such as Nestin, Sox2, and RC2 after injury [172,173]. An NSC might be a type of astrocyte; glial scar formation after injury may partly be due to activated astrocyte-like NSCs differentiating into astrocytes under the control of post-stroke upregulated CNTF [174]. GLAST-positive reactive astrocytes could dedifferentiate and form multipotent spheres in culture following brain stab injury; the results indicated that reactive astrocytes appear to have greater plasticity [172]. Sonic hedgehog (Shh) signaling is reported to be both necessary and sufficient to promote the proliferation of astrocytes in vivo and neurosphere formation in vitro [175]. Cortical reactive astrocytes isolated from the peri-infarct area after stroke can dedifferentiate into neural sphere-producing cells (NSPCs) that possess self-renewal and multipotent ability. Presenilin-1-based Notch 1 signaling is involved in the generation, proliferation, and self-renewal of NSPCs, which is similar to typical NSCs [176]. However, transplanted NSPCs could only differentiate into astrocytes and oligodendrocytes but not neurons in vivo [176]. Thus, reactive astrocytes appear to have greater plasticity to provide a source of multipotent cells or a cellular target for regenerative medicine. 

Recent studies focused on exploring how could astrocytes be redirected into a neuronal lineage. Cultured astrocytes transfected with neuronal transcription factor NeuroD1 could be converted to neurons marked by reduced proliferation, adopted neuronal morphology, expressed neuronal/synaptic markers, and even detected action potentials. Reactive glial cells in the glial scar can be reprogrammed into functional neurons with NeuroD1, a single neural transcription factor, in the stab-injured adult mouse cortex [177]. Reprogramming astrocytes with NeuroD1 after stroke reduced astrogliosis and restored interrupted cortical circuits and synaptic plasticity [178]. Furthermore, a combination of multiple transcriptional factors, ASCL1, LMX1B, and NURR1, as well as another single transcriptional factor, Sox2, can convert reactive astrocytes to neuroblasts or even neurons [179,180]. Signaling of FGF receptor tyrosine kinase promotes dedifferentiation of nonproliferating astrocytes to NSCs, which can be strongly impaired by interferon-γ through phosphorylation of STAT1 [181]. In addition, removal of the p53–p21 pathway and depletion of the RNA-binding protein PTBP1 also contributes to glia-to-neuron conversion [182]. Thus, utilizing reactive astrocytes as an endogenous cellular source for the generation of neuronal cells to repair damaged brain structures is a promising “astro-therapy” for stroke in the future.

### 3.4. Angiogenesis and BBB Repair: Astrocytes and Endothelial Lineage

Remodeling of ischemic injured tissue is not only driven by neurogenesis and plasticity but also influenced by orchestrated cell–cell signaling of neuronal, glial, and vascular compartments [183]. It is well recognized that post-stroke angiogenesis promotes neurogenesis and functional recovery [184], and vascular repair is also important for restoring blood–brain barrier properties [185]. Astrocytes are tightly involved in these above processes. Chemogenetic ablation of a certain subtype of reactive astrocytes worsens motor recovery by disrupting vascular repair and remodeling after stroke characterized by sparse vascularization, increased vascular permeability, and prolonged blood flow deficits [186]. Stroke induces transcriptional changes associated with vascular remodeling which upregulate genes related to sprouting angiogenesis, vessel maturation, and extracellular matrix remodeling in reactive astrocytes. Reactive astrocytes interact with new vessels in the peri-infarct cortex as shown by in vivo two-photon imaging [186]. 

Astrocytic VEGF and MMPs have biphasic functions depending on their temporal expression after stroke. They increase blood vessel permeability and ischemia-induced damage during the acute phase and enhance angiogenesis and restore BBB functions during neurovascular repair phases [187]. Astrocytes could promote angiogenesis via stimulating the RhoA/ROCK pathway in a co-culture system with BMECs [188]. HMGB1-mediated expression of astrocytic IL-6 also promotes angiogenesis and functional recovery after ischemic stroke [189]. In mice deficient in SorCS2, the ability of astrocytes to release endostatin after stroke was lost, which coincided with impaired angiogenesis [190].

Shh secreted from astrocytes is an important molecule for promoting BBB formation and integrity [191]. OGD-activated astrocytes, co-cultured with endothelial cells, can secrete Shh and promote BBB integrity by upregulating tight junction proteins in vitro [192]. Recombinant Shh induces upregulation of astrocytic angiopoietin-1, which leads to upregulation of tight junction proteins and is necessary for stabilizing new leaky vessels; it finally repairs the tight junction and ameliorates BBB disruption post-stroke in vivo [193]. Selective deletion of Na+/H+ exchanger I (NHE1) in astrocytes activates the Wnt/β-catenin signaling pathway, increases BBB integrity, and promotes cerebral vessel repair after ischemic stroke [194]. 

EPC, a circulating progenitor that can differentiate into the endothelial lineage, is mobilized from bone marrow and peripheral blood to the site of injury after stroke [195]. EPCs can integrate into pre-existing vessels and secreted cytokines and contribute to brain vascular remodeling and functional recovery [196]. We explored their therapeutic efficacy and found that intravenously delivered EPCs can home to the ischemic brain and improve long-term stroke outcomes in focal cerebral ischemia, and CXCL12-mediated signaling was involved in EPC-mediated neuroprotection [197]. Reactive astrocytes in the peri-infarct area can release high mobility group box-1 (HMGB1), which is accompanied by the accumulation of endogenous EPCs. SiRNA suppression of HMGB1 in vivo blocks EPC-mediated neurovascular remodeling during post-stroke recovery [198]. The underlying mechanism included the release of soluble HMGB1 from reactive astrocytes enhancing EPC–endothelial adhesive interactions via β2 integrin-RAGE, which increased the transmigration of EPCs across the targeted brain endothelium [199]. Endothelial cells derived from EPCs could acquire several BBB phenotypic characteristics, including expression of tight junction proteins, glucose transporter GLUT-1, and active efflux transporter P-gp through co-culture with astrocytes. They could also obtain functional characteristics such as low permeability to large molecules [200]. The role of EPCs in BBB repair and how reactive astrocytes affect this process still need further exploration.

### 3.5. Oligodendrogenesis and Remyelination: Astrocyte and Oligodendrocyte Lineage

Ischemic stroke can induce white matter injury and result in cognitive and sensorimotor deficits. OPCs are myelin-forming cells contributing to white matter repair. New myelinating oligodendrocytes are necessary for functional recovery [201]. Astrocyte damage leads to oligodendrocyte death and demyelination in primary astro-cytopathies such as Alexander disease and vanishing white matter disease. Astrocyte death is observed prior to oligodendrocyte loss in osmotic demyelination syndrome. Demyelination and astrogliosis are coupled under a neuroinflammatory state in multiple sclerosis [202]. It is also reported that OPCs failed to remyelinate the demyelinated spinal cord in the absence of astrocytes [203]. These findings all suggest the necessary role of astrocytes in oligodendrocyte survival and maturation during injuries. 

Astrocytes share their lineage and interact with oligodendrocytes. Astrocytes express Cx30 and Cx43 which couple to adjacent oligodendrocytes expressing Cx32 and Cx47 by forming heterotypic gap junctions. This physical contact enabling the free flow of small signaling molecules is important in oligodendrocyte maturation and pathology. For example, the absence of Cx47 or Cx32 in oligodendrocytes increased central nervous myelin loss, thus exacerbating clinical outcomes in EAE mice [204]. In addition, pathogenic mutated Cx32 of oligodendrocytes contributes to peripheral demyelination and neuropathy [205]. The detrimental effect of CX loss on remyelination may be attributed to the defective trophic support by astrocytes through gap junctions [206]. Interestingly, recent studies showed that inhibition of astrocytic CX43 channels facilitated the differentiation of OPCs under chronic hypoxia in an astrocyte–OPC co-culture model [207]. 

Astrocytes secrete various inflammatory factors which have a critical role in demyelination diseases, including tumor necrosis factor-α (TNF-α), IL-1β, and interferon-γ (IFN-γ). During ischemic stroke, IL-1β is expressed by astrocytes, which induces oligodendrocyte apoptosis and hypomyelination of periventricular white matter in the hypoxic neonatal brain [208]. Growth factors, including fibroblast growth factor 2 (FGF2) and PDGF, derived from astrocytes control oligodendrogenesis [209]. Most growth factors promote oligodendrogenesis. For example, astrocytes could also secrete BDNF to support the maturation of OPCs during chemical hypoxic stress in vitro and white matter hypoperfusion injury in vivo [210]. In addition, fibrous astrocytes located within white matter constitutively expressed CNTF [211,212], which enhanced the migration of OPCs from SVZ to demyelinated regions [213]. IGF-1 and EPO released from reactive astrocytes in the ischemic brain also enhance oligodendrogenesis after stroke [214]. However, astrocytes tightly control the release of bone morphogenic proteins (BMPs) and prevent maturation of OPCs; BMPs could even induce OPC differentiation into the astrocyte lineage. FGF-2 has been shown to promote OPC proliferation yet inhibit their differentiation to oligodendrocytes [215].

Astrocytes could recruit OPCs to demyelinated zones via secretion of CXCL1, CXCL8, and CXCL10 [216]. The chemokine CXCL12 released by astrocytes acts on OPCs through CXCL12/CXCR4 signaling to induce its proliferation and differentiation in the MS model [217]. In some demyelinating injuries, astrocyte-derived endothelin-1 inhibits remyelination through Notch activation, and this effect can be reversed by a clinically used ET receptor (ET-R) pan-antagonist [218]. Further experiments revealed that the reactive astrocytes regulated the rate of oligodendrocyte regeneration through endothelin-B receptor (ET-B) activation [219]. The OPC maturation is blocked after white matter stroke, which is partly mediated by Nogo receptor 1 (NgR1) signaling; NgR1 antagonist administration after stroke improved post-stroke oligodendrogenesis in a mouse model [220]. Extracellular matrix (ECM) components secreted by astrocytes also affect OPCs. Hyaluronan accumulates in demyelinated lesions in MS, which inhibits OPC maturation but promotes astrocytic differentiation [221]. Another astrocytic ECM factor, laminin, promotes OPC survival and controls their differentiation and migration. 

The subset of astrocytes could also affect oligodendrocyte lineage cells. The reduction of A1-like astrocytes aided OPC maturation and protected against white matter injury under prolonged cerebral hypoperfusion; the underlying mechanism involved mitochondrial migration and Trkβ signaling [222]. However, the relationship between astrocytes and myelin is mainly studied in MS and other inflammatory CNS diseases, more direct evidence of astrocyte–oligodendrocyte crosstalk during ischemic stroke is still needed.

## 4. Conclusions

**Figure 2:**
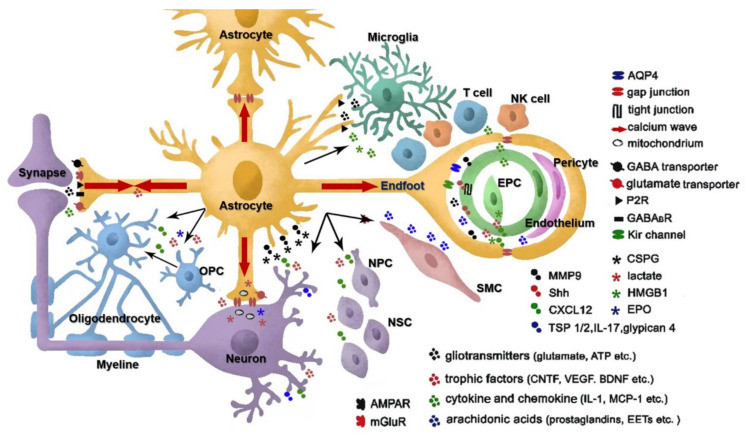
A schematic representation of the diverse functions mediated by astrocytes in a cell–cell interaction perspective. Astrocytes can regulate cerebral blood flow and synaptic transmission through gliotransmitter release. The gap junctions allow for intercellular calcium wave and metabolic substrate propagation. Astrocytes provide neuroprotection in the acute phase of stroke through antioxidation and antiexcitatory effects, metabolic support, and mitochondria transfer through astro-neuronal signaling. However, they also contribute to the pathogenesis of stroke by disrupting blood–brain barrier integrity and aggregating inflammation through interaction with microglia and infiltrating immune cells. Reactive astrocytes can form glial scars to hamper axon growth, but they also interact with stem cells, including OPCs, NSCs, and EPCs, to promote neurogenesis, synaptogenesis, angiogenesis, BBB repair, and even oligodendrogenesis. AMPAR: AMPA subtype glutamate receptor; mGluR: metabotropic glutamate receptor; P2R: purinergic receptor; GABABR: metabolic GABAB receptor; SMC: smooth muscle cell; OPC: oligodendrocyte precursor cell; EPC: endothelial precursor cell; NSC: neural stem cell; NPC: neural progenitor cell.

Despite this central role in brain function, astrocytes have been largely overlooked in the study of stroke pathogenesis and recovery in the past. To date, neurocentric therapeutics have been found to lack efficacy in reducing infarction or improving functional recovery clinically. So, a comprehensive understanding of the astrocytic responses to stroke may be necessary to develop more effective treatment strategies. In this review, we focused on discussing the communication of astrocytes with other cells in the CNS after ischemic stroke, both in the acute phase and in the recovery state, as shown in Figure 2. Reactive astrocytes provide neuroprotection in the acute phase of ischemic stroke through antioxidation and antiexcitatory effects and metabolic support. In the meantime, reactive astrocytes also play a vital role in neuroinflammation and brain edema by communicating with microglia and endothelial cells. Astrocytes form glial scars in the chronic phase and hinder functional recovery. They also contribute to neurorestoration involving neurogenesis, synaptogenesis, angiogenesis, and oligodendrogenesis by crosstalk with stem cells and cell lineage. Astrocytes even have stem cell-related properties themselves and are sources of multipotent cells that may repair damaged brain. The local, regional, temporal, and sexual heterogeneity of reactive astrocytes after stroke is still awaiting further research. New technologies such as transcriptome analysis, optogenetics approaches, and genetic modulation will give more hints on reactive astrocytes’ functions during ischemic stroke. Multivariate and clustering analysis of molecular and functional data will facilitate the future study. Astrocyte-targeting therapies to potentiate astrocytic protective actions and inhibit detrimental ones, as well as to restore their homeostatic, modulatory, and defensive functions, are very attractive and awaking further exploration. 

## Data Availability

Not applicable.

## Abbreviations

Aquaporin 4 = AQP4; archaerhodopsins = Arch; blood–brain barrier = BBB; bone morphogenic proteins = BMPs; C-terminal = CT; C-X-C motif ligand = CXCL; calcium-translocating channelrhodopsin = CatCh; cerebral blood flow = CBF; central nervous system = CNS; channelrhodopsin-2 = ChR2; halorhodopsin = NpHR; chondroitin sulfate proteoglycans = CSPGs; ciliary neurotrophic factor = CNTF; ciliary neurotrophic factor = CNTF; complement component subunit 1q = C1q; connexins = CXs; epidermal growth factor = EGF; epoxyeicosatrienoic acids = EETs; erythropoietin = EPO; endothelin-B receptor = ET-B; ET receptor = ET-R; experimental autoimmune encephalomyelitis = EAE; fibroblast growth factor 2 = FGF2; glial derived neurotrophic factor = GDNF; glial fibrillary acidic protein = GFAP; high mobility group box-1 = HMGB1; hypoxia-inducible factor-alpha = HIF-1a; inducible nitric oxide synthase = iNOS; interferon-γ = IFN-γ; interlukin-1β = IL-1β; lipopolysaccharide = LPS; chemoattactant protein-1 = MCP-1; matrix metalloproteinase-9 = MMP-9; microRNAs = miRNAs; non–rapid eye movement sleep = NREM; nuclear factor κB = NF-κB; NOD-like receptors = NLRs; natural killer cells = NKs; neural sphere-producing cells = NSPCs; neural progenitor cells = NPCs; neural stem cells = NSCs; Na+/H+ exchanger I = NHE1; Nogo receptor 1 = NgR1; orosomucoid-2 = ORM2; pattern recognition receptors = PRRs; rapid eye movement sleep = REM; Sonic hedgehog = Shh; secreted protein, acidic and rich in cysteine = SPARC; subventricular zone = SVZ; transforming growth factor-beta = TGF-β; toll-like receptors = TLRs; thrombospondin = TSP; tumor necrosis factor-α = TNF-α; tight junctions = TJs.
